# (Un)expected Similarity of the Temporary Adhesive Systems of Marine, Brackish, and Freshwater Flatworms

**DOI:** 10.3390/ijms222212228

**Published:** 2021-11-12

**Authors:** Philip Bertemes, Robert Pjeta, Julia Wunderer, Alexandra L. Grosbusch, Birgit Lengerer, Kevin Grüner, Magdalena Knapp, Birte Mertens, Nikolas Andresen, Michael W. Hess, Sara Tomaiuolo, Armin Zankel, Patrik Holzer, Willi Salvenmoser, Bernhard Egger, Peter Ladurner

**Affiliations:** 1 Institute of Zoology, University of Innsbruck, 6020 Innsbruck, Austria; philip.bertemes@uibk.ac.at (P.B.); robert.pjeta@innpath.at (R.P.); julia.wunderer@gmail.com (J.W.); alexandra.grosbusch@uibk.ac.at (A.L.G.); birgit.lengerer@uibk.ac.at (B.L.); kevin.gruener@student.uibk.ac.at (K.G.); magdalena.knapp@student.uibk.ac.at (M.K.); birte.mertens@i-med.ac.at (B.M.); andresen.n13@gmail.com (N.A.); patrik.holzer@tirol-kliniken.at (P.H.); willi.salvenmoser@uibk.ac.at (W.S.); bernhard.egger@uibk.ac.at (B.E.); 2 Center of Molecular Bioscience Innsbruck, University of Innsbruck, 6020 Innsbruck, Austria; 3 Institute of Molecular Biology, Innsbruck Medical University, 6020 Innsbruck, Austria; 4 Institute of Histology and Embryology, Innsbruck Medical University, 6020 Innsbruck, Austria; Michael.Hess@i-med.ac.at; 5 Zoonoses of Animals Unit, Veterinary Bacteriology, Infectious Diseases in Animals Scientific Directorate, Sciensano, 1050 Brussels, Belgium; Sara.Tomaiuolo@UGent.be; 6 Institute of Electron Microscopy and Nanoanalysis, NAWI Graz, Graz University of Technology, Steyrergasse 17, 8010 Graz, Austria; armin.zankel@felmi-zfe.at; 7 Graz Centre for Electron Microscopy, Steyrergasse 17, 8010 Graz, Austria

**Keywords:** *Macrostomum*, non-permanent adhesion, glue, aquatic, duo-gland adhesive system, RNA interference, in situ hybridisation

## Abstract

Many free-living flatworms have evolved a temporary adhesion system, which allows them to quickly attach to and release from diverse substrates. In the marine *Macrostomum lignano*, the morphology of the adhesive system and the adhesion-related proteins have been characterised. However, little is known about how temporary adhesion is performed in other aquatic environments. Here, we performed a 3D reconstruction of the *M. lignano* adhesive organ and compared it to the morphology of five selected *Macrostomum*, representing two marine, one brackish, and two freshwater species. We compared the protein domains of the two adhesive proteins, as well as an anchor cell-specific intermediate filament. We analysed the gene expression of these proteins by in situ hybridisation and performed functional knockdowns with RNA interference. Remarkably, there are almost no differences in terms of morphology, protein regions, and gene expression based on marine, brackish, and freshwater habitats. This implies that glue components produced by macrostomids are conserved among species, and this set of two-component glue functions from low to high salinity. These findings could contribute to the development of novel reversible biomimetic glues that work in all wet environments and could have applications in drug delivery systems, tissue adhesives, or wound dressings.

## 1. Introduction

Bioadhesion can be found across many phyla throughout the tree of life [1,2,3]. Different strategies evolved to perform such an important task in terrestrial environments, e.g., mechanical adhesion through little hooks such as in the plant *Arctium* [4], biofilms produced by bacteria to provide a habitat in otherwise unsuitable terrain [5], adhesion relying on the physical properties of the van der Waals forces is used by the gecko [6], or silks produced by spiders and other arthropods [7]. In the aquatic environment, many species have developed glues to anchor the animal permanently to the substrate in rough marine conditions, e.g., mussels, which rely on a modified amino acid tyrosine (3,4-dihydroxyphenyl-L-alanine; DOPA) in a very low pH environment [8,9,10]. In contrast to these permanently adhering animals, many aquatic invertebrates, such as echinoderms, flatworms, and limpets, evolved a non-permanent adhesion system [11,12,13]. The glues of non-permanent adhering animals are mainly composed of proteins and carbohydrates in varying proportions, and no DOPA has been reported so far (reviewed in [14]). Free-living flatworms are intriguing model systems to study non-permanent adhesion, as they occur in diverse aquatic habitats, are easy to culture, and many molecular biology tools are available for selected species.

*Macrostomum* is a genus that comprises animals from the early-branching order Macrostomorpha within the free-living flatworms (‘Turbellaria’) of the phylum Platyhelminthes [15]. They are small (in the millimeter range) bilaterian animals that occur only in wet environments. Most macrostomid species inhabit marine environments, but also brackish and freshwater species are common. About 180 species are currently described, and new species are continuously added [16,17,18,19,20,21,22]. Despite being a quite diverse genus, a single species is highlighted in many different research fields—*Macrostomum lignano*. This species was used as a model organism for stem cell research, regeneration, ageing, embryonic development, the evolution of sexual reproduction, and bioadhesion (reviewed in [23]). *M. lignano* occurs in the interstitial zone, more specifically in the upper few centimeters of the substrate on a marine coast. It is able to rapidly adhere to and release from a substrate in seawater using a duo-gland adhesive system [13]. The morphology of the duo-gland adhesive system of *M. lignano* was described in detail by Lengerer et al. [24]. Briefly, about 130 adhesive organs, each composed of three cells (one adhesive, one releasing, and one anchor cell), are distributed in a horseshoe-shaped manner on the ventral side of the tail plate. For attachment, the animals secrete adhesive proteins that strongly adhere the tail of the animals to the substrate. For detachment, the animals secrete a releasing substance from their releasing gland cells.

Two large adhesive proteins, “*Macrostomum lignano* adhesion protein 1” (Mlig-ap1; 5407 amino acids) and “*Macrostomum lignano* adhesion protein 2” (Mlig-ap2; 14,794 amino acids), were identified in the vesicles of the adhesive gland cell. Strikingly, the simultaneous knockdown of *Mlig-ap1* and *Mlig-ap2* leads to animals without adhesive vesicles, confirming that these two proteins are the major component of the secreted glue [13]. Mlig-ap2 attaches to the substrate, and Mlig-ap1 has a cohesive function that mediates the connection between the surface-bound Mlig-ap2 and the microvilli of the anchor cells of the animal. Mlig-ap1 contains a core region of known protein–protein and protein–carbohydrate-binding domains, while two-thirds of this protein is composed of lysine–arginine-rich repetitive regions on the C- and N-terminal ends. The second protein, Mlig-ap2, contains the protein–protein interaction domains in its C- and N-terminal ends, while its central part is composed of two large repeat regions (21 repeats of a 255 aa long sequence, followed by 25 repeats of a 221 aa long sequence). Mlig-ap2 is glycosylated and can be labelled by the sugar-binding lectin peanut agglutinin (PNA) [13]. It was shown in *M. lignano* that an RNA interference (RNAi) knockdown of either *Mlig-ap1* or *Mlig-ap2* led to a non-adhesive phenotype [13]. Furthermore, it was proposed that a yet unidentified molecule secreted by the releasing gland cell induces detachment between Mlig-ap1 and the microvilli of the animal. After detachment, footprints consisting of Mlig-ap1 and Mlig-ap2 remain on the substrate. Knowledge on bioadhesive molecules in other *Macrostomum* species is scarce, but in the freshwater *M. poznaniense*, a knockdown of the *ap2-like* gene also impeded attachment [25].

In addition to the secreted adhesive proteins, the morphological integrity of the anchor cells is essential for the adhesion capacity in *M. lignano* [26,27]. An anchor cell-specific intermediate filament-like protein was identified in the tail of *M. lignano* [26]. This “*Macrostomum lignano* intermediate filament 1” (Mlig-if1) protein was considered to transmit the adhesive forces within the anchor cell from the microvilli to the extracellular matrix via hemidesmosomes. *Mlig-if1* knockdown with RNAi led to animals with altered anchor cell morphology that were unable to attach to the substrate.

With this work, we explored whether the ecological conditions of the habitat affect the morphology of the adhesive system and the proteins involved in the adhesion of different macrostomid species. Especially, we want to address the question of whether bioadhesion of a marine species is comparable to adhesion in brackish water or freshwater. A prerequisite for future biomimetic manufacturing of flatworm-based holdfasts is a detailed understanding of the organisation of an adhesive organ. Here, we performed a 3D reconstruction of an adhesive organ of *M. lignano* and compared the morphology to five macrostomid species from different environments: the two marine species including *Macrostomum spirale* Ax 1956 [28] and *Macrostomum pusillum* Ax 1956 [28], the brackish water species *Macrostomum hystrix* Ørsted 1843 [29] *sensu* Luther 1905, and the two freshwater species *Macrostomum poznaniense* Kolasa 1973 [30] and *Macrostomum tuba* von Graff 1882 [31]. We compared adhesion-related proteins, including their domain organisations, expression patterns, and functions. Overall, the work could contribute to a future generation of synthetic adhesives tailored for diverse aquatic environments or applications on or inside the human body.

## 2. Results

### 2.1. General Overview of Adhesion Organs in Macrostomum

**Figure 1:**
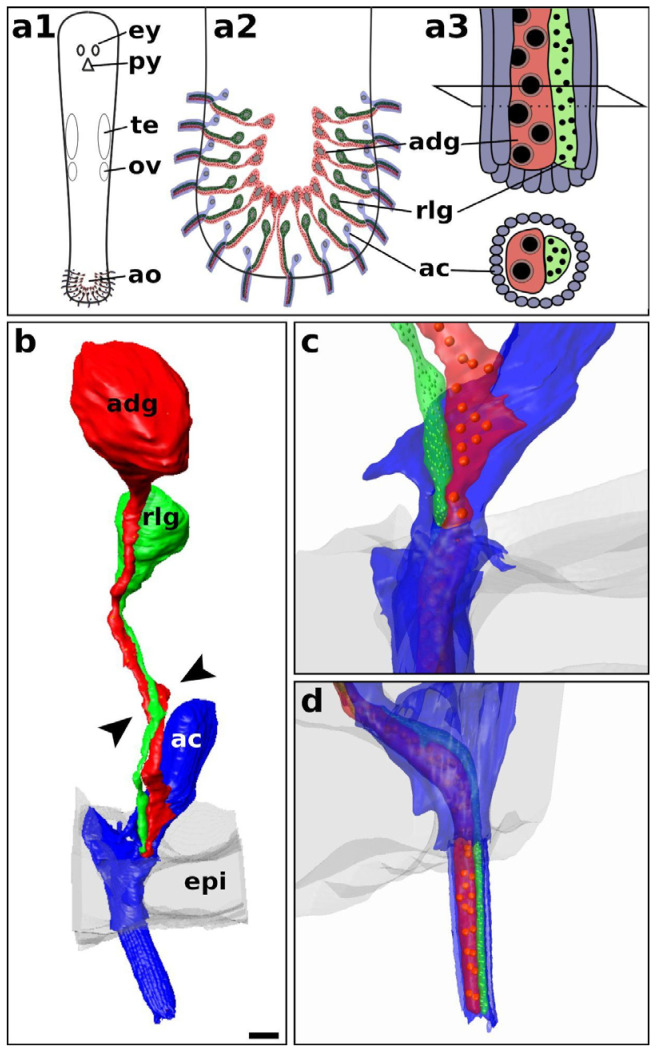
Schematics of the adhesive organ organisation and 3D reconstructed model. Schematics of the hierarchical localisation and organisation of adhesive organs of an adult *Macrostomum* (**a1**–**a3**). Three-dimensional reconstruction of a complete adhesive organ in an adult *Macrostomum lignano* (**b**). The necks of the adhesive and releasing gland cell penetrate the anchor cell (**c**). The microvilli collar of the anchor cell around both gland cell necks forms the adhesive papilla (**d**). Adhesive gland cell (adg, red), anchor cell (ac, blue), adhesive organ (ao), epidermal cell (epi, grey), eyes (ey), ovaries (ov), pharynx (py), testes (te), releasing gland cell (rlg, green). Arrowheads in (**b**) show the twisting of the gland necks. Adhesive (red) and releasing (green) vesicles were schematically drawn into the reconstruction. Scale bar in (**b**) is 2 µm.

We observed that *Macrostomum* species occurring in marine, brackish water, and freshwater environments all possess a highly similar organisation of the adhesive system. The hierarchical organisation is shown in Figure 1. The adhesive system is located at the ventral side of the tail of the animals (Figure 1(a1)) and comprises around one hundred adhesive organs in a horseshoe-shaped pattern (Figure 1(a2)). Each adhesive organ is built by three different cell types: (1) an adhesive gland cell (red in images), a releasing gland cell (green in images), and a modified epidermal cell called anchor cell (blue in images). The gland necks of the adhesive and releasing cells run in close proximity and insert into the anchor cell at an oblique angle. At the external part of the adhesive organ, termed as adhesive papilla, the microvilli of the anchor cell form a collar around the gland cell necks. The cell bodies of the adhesive and the releasing gland cells are located towards the centre of the tail plate. The adhesive gland cell contains vesicles with adhesive proteins. The releasing gland cell contains smaller vesicles with a releasing substance (Figure 1(a3)).

We generated a stack of scanning electron microscope images from an *M. lignano* tail region consisting of 708 individual sections. From a subset containing a total of 441 images (data can be retrieved from an online repository, see Materials and Methods), we reconstructed a 3D model from the adhesive gland cell, the releasing gland cell, and the anchor cell (Figure 1b–d, Supplementary Video S1). Here, the adhesive gland cell body was situated anteriormost within the tail plate. The cell body of the releasing gland cell was adjacent to the gland neck of the adhesive gland cell. The nucleus of the anchor cell was located recessed below the basement membrane, whereas the cell body was flattened and expanded to increase the contact surface to the basal membrane (Supplementary Video S1). In the observed sample, the necks of the adhesive and releasing gland cell ran in a twisted way towards the anchor cell (Figure 1b arrowheads, Supplementary Video S1). They penetrated the cell body of the anchor cell from the side (Figure 1c). In the adhesive papilla, the tips of the gland cell necks were surrounded by the collar of microvilli of the anchor cell (Figure 1d).

### 2.2. Morphology of Adhesion Organs in Different Macrostomum Species

**Figure 2:**
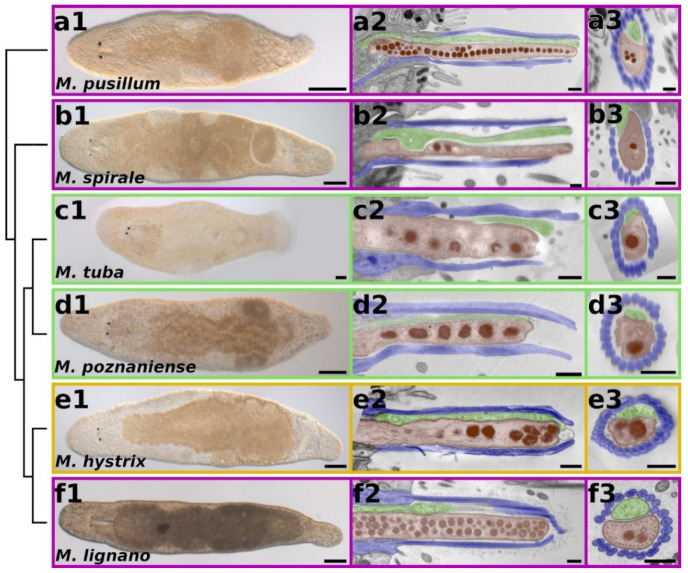
Morphology of the adhesive systems of six *Macrostomum* species. Animals are positioned according to the phylogenetic relationships. Differential interference contrast (DIC) images of adult live squeeze preparations of the representatives of this study (**a1**–**f1**). Transmission electron microscopic (TEM) images of sagittal sections (**a2**–**f2**) and cross sections (**a3**–**f3**) of the adhesive organs. Adhesive gland cell necks (highlighted in red); releasing gland cell necks (highlighted in green); microvilli of the anchor cell (highlighted in blue). Phylogenetic relationships adapted from [16]. Marine species outlined in pink, brackish species outlined in yellow, freshwater species outlined in green. Scale bars: 100 µm for DIC images, 500 nm for TEM images. Samples displayed in panels (**f2**,**b3**,**f3**) were high pressure frozen and freeze-substituted; (**a2**,**a3**,**b2**) were fixed according to Eisenmann and Alfert; (**c2**–**e2**) and (**c3**–**e3**) were fixed according to Rombout (see Section 4 for details).

Knowledge about the interrelationships of the species studied in this work is a prerequisite to infer potential ancestral or derived characters (Figure 2). The marine species *M. pusillum* forms the sister group of all other represented *Macrostomum* species, followed by the also marine *M. spirale*, which is the adelphotaxon of the remaining species, where the freshwater-dwelling animals (*M. tuba* and *M. poznaniense*) are the sister group of the brackish *M. hystrix* and the marine *M. lignano* (adapted from [16]). The three marine (pink in figures), the brackish (yellow in figures), and the two freshwater species (green in figures) (Figure 2(a1–f1)) showed a high similarity in the morphology of the adhesive organ in sagittal sections. They all held a single adhesive gland cell neck and a single releasing gland cell neck, which were surrounded by the microvilli of a single anchor cell (Figure 2(a2–f2)). Notably, the cell bodies of the adhesive gland cells in *M. pusillum* were located further anterior in the tail plate compared to the adhesive gland cell bodies of the others. The microvilli collar around the gland cell necks was evident in cross sections of the adhesive papilla (Figure 2(a3–f3)). In the adhesive papilla, the adhesive gland neck was located ventrally to the releasing gland neck in all species. The adhesive vesicles had an electron-dense inner core and a lighter outer rim. The nitrogen content was analysed by electron energy loss spectroscopy (EELS) and electron spectroscopic imaging (ESI) in *M. pusillum*, *M. hystrix*, and *M. tuba* and reflects the density of protein in the respective morphological structure. This analysis revealed a high nitrogen content in the dark core of the adhesive vesicles of all three species (Supplementary Figure S2). These observations indicate that the diverse aquatic habitats did not lead to a significant alteration of the morphology of the adhesive organs of the studied species. However, preliminary approximate measurements of the size of adhesive vesicles were about 300 nm in *M. lignano* and *M. hystrix* and about 400 nm in *M. tuba.* The adhesive granules’ size, the relative proportion of the ap1-core and ap2-rim, and possible substructures will be investigated in the future.

### 2.3. Conservation of Protein Domains in Adhesives

**Figure 3:**
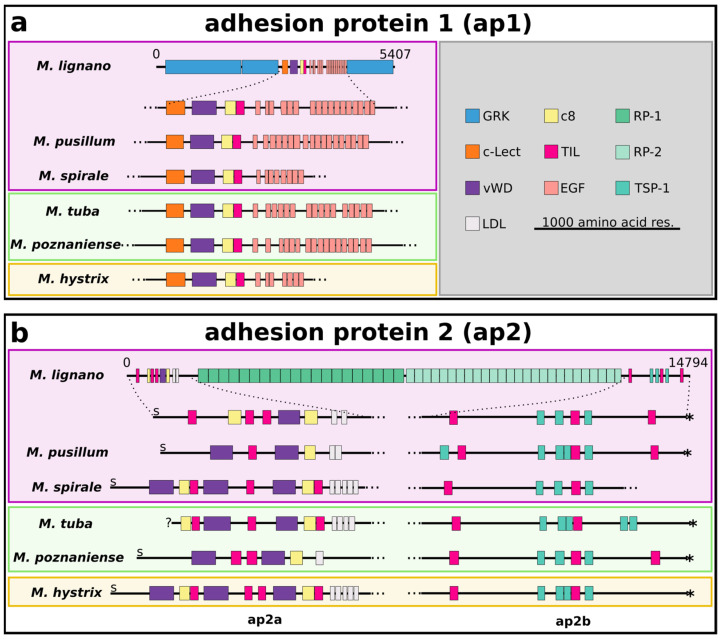
Protein domain architecture of ap1- and ap2-like adhesive proteins. Protein domain structure of Mlig-ap1-like (**a**) and Mlig-ap2-like (**b**) proteins in six *Macrostomum* species. C8, domain of eight conserved cysteines; c-Lect, c-type lectin domain; EGF, epidermal growth factor-like domain; GRK, low complexity region containing a high amount of arginine, lysine, and glycine; LDL, low-density lipoprotein receptor-like domain; RP-1, repeat motif 1; RP-2, repeat motif 2; TIL, trypsin inhibitor-like domain; TSP-1, thrombospondin 1-like domain; vWD, von Willebrand factor type D-like domain. S in the N-terminal end represents a signal peptide. Marine species outlined in pink, brackish species outlined in yellow, freshwater species outlined in green. The asterisk in the C-terminal end represents a stop codon. Dotted lines indicate a non-shown protein region. The 5′-end of Mtub-ap1 was probably not completely assembled and is marked by a question mark. The scale bar is true for the closed-up protein fragments and not the whole proteins of *M. lignano*.

We analysed whether the marine, brackish, or freshwater habitat had an impact on the protein domain architecture of the adhesive proteins. We generated a de novo transcriptome for *M. tuba* (see Materials and Methods for details). A total of 271,070 Trinity “transcripts” and 155,775 Trinity “genes” were assembled with a GC content of 51.67%. A BUSCO (Benchmarking sets of Universal Single-Copy Orthologs) analysis with 954 core metazoan genes reported the following transcriptome completeness: 85.7% (Single: 9.6%, Duplicated: 76.1%), Fragmented 2.9%, Missing 11.4%. Of all the assembled transcripts, 90,165 or 83,324 transcripts were annotated with a blastx or a blastp hit against the UniProt Swiss-Prot database, respectively. A total of 24,020 transcripts had a transmembrane helix–loop–helix (TmHMM) signature, and 11,086 transcripts were found to have a sequence coding for a signal peptide. Published transcriptomes of the other species were downloaded. Using BLAST search, we identified *ap1-like* and *ap2-like* sequences (Figure 3; Supplementary Figure S3).

### 2.4. Adhesion Protein 1

Mlig-ap1 contained large repetitive amino acid stretches composed of glycine, arginine, and lysine, “GRK”, in the N- and C-terminal region, while the core region was composed of a motif of known protein domains (Figure 3a). In contrast to *M. lignano*, only short stretches of the “GRK” (or “SRK” in *M. pusillum*) enriched regions were found on the N- and C-terminal ends in the other five *Macrostomum* species. However, we identified Mlig-ap1 protein homologues with high similarity in all five *Macrostomum* species exclusively in the core region (Supplementary Figure S3). In general, the core region was consistently composed of a C-type lectin domain followed by a von Willebrand factor D type domain (vWD), a domain of eight conserved cysteines (C8), and a directly adjacent trypsin inhibitor-like domain (TIL). Additionally, a variable amount of EGF-like domains were present in all species. In *M. pusillum*, Mpus-ap1 contained 18 EGF domains. Mspi-ap1 in *M. spirale* contained eight EGF domains; however, this assembled transcript was shorter than for the other species. The freshwater species *M. tuba* and *M. poznaniense* had 16 and 17 EGF domains present in Mtub-ap1 and Mpoz-ap1, respectively. Finally, the brackish species *M. hystrix* showed only seven EGF domains, but Mhtx-ap1 was most probably not assembled completely in the transcriptome.

### 2.5. Adhesion Protein 2

The published sequence of Mlig-ap2 (Figure 3b) [13] contains a conserved protein domain-rich region in the N-terminal end, encompassing TIL, vWD, C8, and low-density lipoprotein (LDL) domains, and one region in the C-terminal end, containing TIL domains and Thrombospondin type 1 domains (TSP-1). The core region of Mlig-ap2 is composed of two highly repetitive protein motifs (RP-1 and RP-2) [13]. Likewise, Mpoz-ap2 was assembled with two repeat motifs in the centre of the protein [25]. However, short-read-based transcriptome assembly was not able to fully assemble the long ap2, including repeat regions 1 and 2, in the other species. Therefore, all ap2 homologues remained separated into an N-terminal region, termed as -ap2a, and a C-terminal domain region, termed as -ap2b (after their species-specific prefix). The C-terminal part of ap2a contained a partial repeat motif 1 in *M. pusillum* and in *M. tuba*, and the N-terminal region of ap2b contained partial or multiple repeat units of motif 2 in *M. pusillum*, *M. spirale*, *M. hystrix*, and *M. tuba*. The general structure of ap2a and ap2b was highly similar in the studied *Macrostomum* species (Supplementary Figure S3). ap2a always had two vWD domains interposed by either one (*M. pusillum*, *M. spirale*, and *M. tuba*) or two (*M. poznaniense* and *M. hystrix*) TIL domains and followed by a single C8 domain. The number of LDL domains at the end of the N-terminal regions varied between one and five. *M. spirale*, *M. tuba*, and *M. hystrix* showed an additional TIL domain in front of the LDL domains. Furthermore, *M. tuba* featured an additional TIL and C8 domain at the beginning of the N-terminal region, while *M. spirale* and *M. hystrix* contained an additional vWD, TIL, and C8 domain at the beginning of the N-terminal region. Generally, ap2b was similar to the C-terminal region of Mlig-ap2 containing two TIL domains interposed by two (*M. poznaniense* and *M. spirale*) or three (*M. pusillum*, *M. tuba*, and *M. hystrix*) TSP-1 domains. This part was followed by one TSP-1 domain except for *M. tuba,* where two TSP-1 were present. Finally, *M. pusillum* and *M. poznaniense* showed an additional TIL domain at the end of the C-terminal region.

In *M. lignano*, PNA lectin staining has revealed the presence of the sugar galactosyl (β-1,3) N-acetylgalactosamine (Gal-β(1–3)-GalNAc) in the outer rim of the adhesive vesicles [24]. It was later shown that this sugar is part of the glycosylated Mlig-ap2 [13]. In *M. hystrix* and *M. pusillum*, PNA staining likewise detected this sugar in the outer rim of the adhesive vesicles (see Supplementary Figure S4). Notably, PNA did not stain the adhesive system of the freshwater species *M. tuba.*

### 2.6. Anchor Cell-Specific Intermediate Filaments in Macrostomum Species

**Figure 4:**
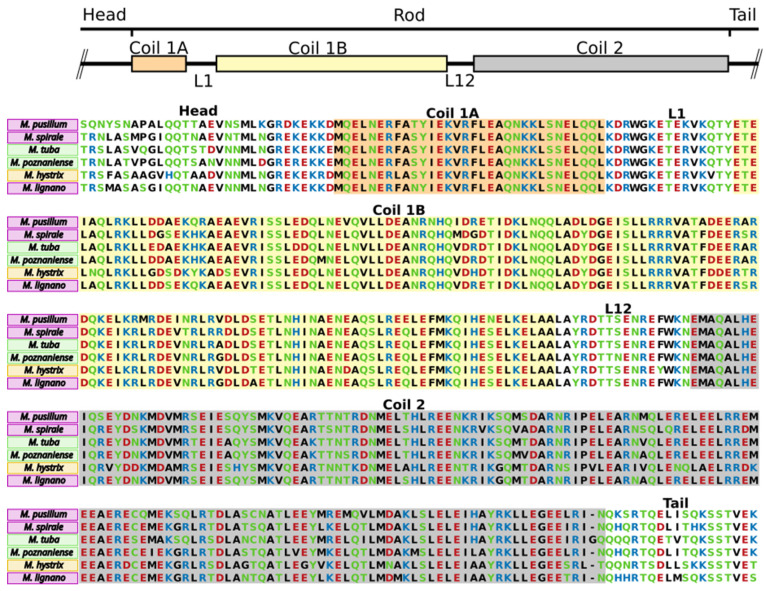
Conservation of the anchor cell-specific intermediate filament between six *Macrostomum* species. Alignment of Mlig-if1-like protein to its homologues in *M. pusillum*, *M. spirale*, *M. tuba*, *M. poznaniense*, and *M. hystrix*. Marine species outlined in pink, brackish species outlined in yellow, freshwater species outlined in green. The amino acid residues are coloured by their polarity: green neutral+polar; black neutral+non-polar; blue basic+polar; red acidic+polar). Coil1 is divided into Coil1A (highlighted in orange) and Coil1B (highlighted in yellow), linked by the linker region L1. Coil2 (highlighted in grey) is connected to Coil1B with the linker region L12.

In *M. lignano*, the anchor cell-specific intermediate filament Mlig-if1 is essential for attachment [26]. We analysed whether these intermediate filaments were present in the other *Macrostomum* species of this study. It was previously shown that Mlig-if1 contained all characteristic intermediate filament protein domains, including a head, rod, and tail domain [26]. Using BLAST search, we identified homologues of the Mlig-if1 protein in the transcriptomes of the investigated species and amplified and sequenced them (for primers, see Supplementary Table S5). These intermediate filament-like transcripts showed very high similarity to Mlig-if1 and also included the characteristic regions (Figure 4). The rod region was composed of three coil domains (Coil 1A, Coil 1B, Coil 2) and two linkers L1 and L12. Both linkers of all investigated species were identical on an amino acid level to the Mlig-if1-like counterpart, except for *M. hystrix*, where one amino acid residue was different on both L1 and L12, and *M. poznaniense*, where one amino acid residue was different in L12 (Figure 4). From this finding, it is very likely that the anchor cell-specific intermediate filaments play an identical role in marine, brackish water, and freshwater adhesion.

### 2.7. Adhesion Assay in Different Salt Concentrations

We assessed if salt concentration levels influenced the attachment of freshwater, brackish water, and seawater *Macrostomum* representatives (Table 1). The freshwater species *M. tuba* was able to attach in salt concentrations of up to 2 parts per million (ppm), and animals started to dissolve at 5 ppm. The brackish water species *M. hystrix* was able to attach in a range between 2 ppm and up to 35 ppm and started to stop moving at 45 ppm. The marine species *M. pusillum* and *M. spirale* were able to adhere at salt concentration levels between 2 ppm and 60 ppm.

### 2.8. In Situ Hybridisation of ap1 and ap2

**Figure 5:**
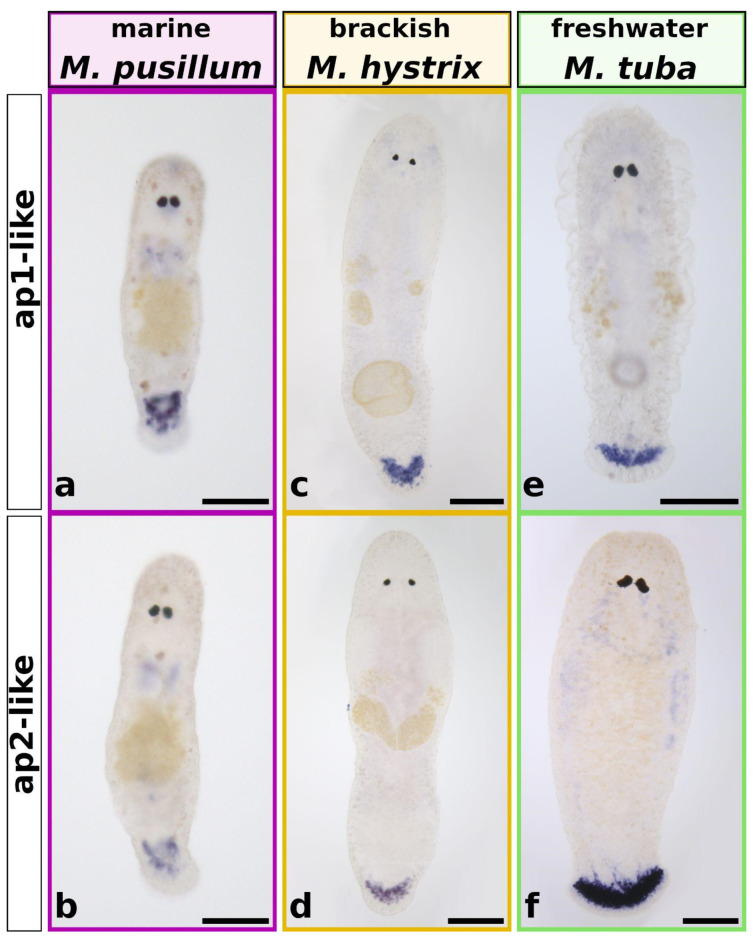
Whole-mount in situ hybridisation of *ap1*- and *ap2-like* genes in three *Macrostomum* species. *ap1* and *ap2* expression in the marine *M. pusillum* (**a**,**b**), in the brackish water *M. hystrix* (**c**,**d**), and in the freshwater *M. tuba* (**e**,**f**). Marine species outlined in pink, brackish species outlined in yellow, freshwater species outlined in green. Scale bars: 50 µm in (**a**,**b**); 100 µm in (**c**–**f**).

Next, in situ hybridisation (ISH) of the *Mlig-ap1-like* and the *Mlig-ap2-like* transcripts was performed for all species. We selected one marine (*M. pusillum*), one brackish water (*M. hystrix*), and one freshwater (*M. tuba*) representative. The in situ hybridisations of *ap1* and *ap2* of *M. lignano* and *M. poznaniense* were already published, and ISH failed in *M. spirale*. All three here analysed species exhibited an ISH expression pattern exclusively in the tail region. As the cell bodies of the adhesive gland cells in *M. pusillum* were located further anterior in the tail plate, the ISH pattern was also found more anterior (Figure 5a,b) compared to *M. hystrix* (Figure 5c,d) and *M. tuba* (Figure 5e,f). There, the adhesive gland cell bodies were accumulated closer to the distal tip of the tail and were arranged in a broad and slightly bent stripe. Control ISH with the respective sense probes showed no staining for both probes in the three species (Supplementary Figure S6). Based on these findings, *ap1-like* and *ap2-like* gene expression was exclusively observed in the tail plate of the three macrostomid species. No obvious alteration in the localisation of gene expression tied to the environmental conditions was found.

### 2.9. RNAi Knockdown of Adhesion Protein 1 and 2 Genes

**Figure 6:**
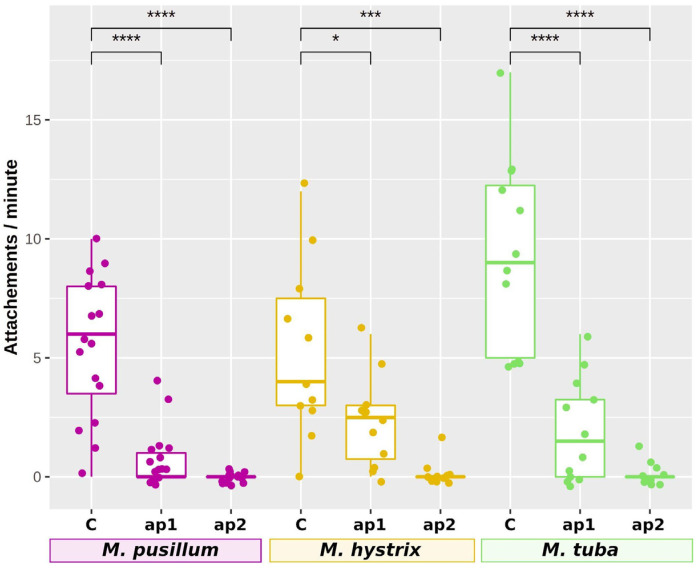
Adhesion assay of RNAi-treated animals. Attachments per minute as observed by different investigators of control animals, *ap1* RNAi-, and *ap2* RNAi-treated animals. Marine species outlined in pink, brackish species outlined in yellow, freshwater species outlined in green. The significance of a pairwise t.test is shown above the box plots (* = *p*-value < 0.5; *** = *p*-value < 0.001; **** = *p*-value < 0.0001). C: control, animals were not treated with RNAi; *ap1*: RNAi against *ap1-like*; *ap2*: RNAi against *ap2-like* transcript.

The function of Mlig-ap1-like and Mlig-ap2-like proteins was explored in the three species from our ISH experiments. Here, we showed that a knockdown of *ap1*- and *ap2-like* genes in the marine *M. pusillum,* the brackish water *M. hystrix*, and the freshwater *M. tuba* resulted in animals with a reduced capacity of attachment in *ap1* knockdown, or a complete loss of attachment in *ap2* knockdown (Figure 6, raw data are available in supplementary file in an online repository at doi:10.5281/zenodo.5519227). Independent from the aquatic habitat, these findings confirm the essential role of the adhesive proteins in the studied species.

## 3. Discussion

### 3.1. Macrostomum Adhesion Organ Morphology Is Not Influenced by the Habitat

In macrostomid flatworms, adhesion is realised by a duo-gland adhesive system consisting of adhesive organs. The distribution and the number of adhesive organs vary between species. Within the Macrostomorpha, adhesive organs can be found along the entire length of the whole body, for example, in *Haplopharynx* sp. or in *Bradynectes* sp. [32]. Furthermore, adhesive organs were also described to be located at the dorsal body region in *Microstomum* sp. and *Paromalostomum* sp. [32]. In the genus *Macrostomum*, the adhesive system is composed of 100 to 300 individual adhesive organs [33]. They are distributed in a stripe at the ventral side of the tail plate. This stripe can consist of either one single or multiple rows of individual adhesive organs. The canonical composition of a single adhesive organ involves only three cell types: adhesive and releasing gland cells and the anchor cell [32]. A common feature of the *Macrostomum* taxon is that an adhesive organ consists of one cell of each cell type. In addition, adhesive and releasing gland cell necks share a collar of enforced microvilli. In other congeners, the same pattern was described in different habitats: in many limnic *Macrostomum* species [17,19,25,34,35], brackish water species [21,22,36], and marine species [18]. Here, we compared six representative marine, brackish, and freshwater *Macrostomum* species. We could not observe any difference in adhesive organ localisation or structure. The reconstructed 3D model of the adhesive organ in *M. lignano* is therefore true for many other *Macrostomum* species.

### 3.2. The Two Adhesive Proteins ap1 and ap2 Are Present in All Investigated Species

*M. lignano* is a marine species, but it has been shown that it can adhere to the substrate from very low (2 parts per million) to high salinity (60 ppm) conditions [13]. We showed here that the adhesive capacity is unaffected by salinity in other *Macrostomum* species. This could mean that the adhesive system is functionally the same and might be independent of environmental charge-based interactions. The glue consists of the two large adhesive proteins Mlig-ap1 and Mlig-ap2. The two proteins are segregated within the adhesive vesicles, with Mlig-ap1 forming an electron-dense inner core and Mlig-ap2 a lucid outer rim [13]. We show that this separation in two distinct vesicle areas was present in all investigated species, regardless of their habitat. Furthermore, homologous sequences for ap1 and ap2 were found in all representatives of marine, brackish, and freshwater environments. With in situ hybridisation, it was shown that *Mlig-ap1* and *Mlig-ap2* adhesive genes are expressed exclusively in the adhesive organs of *M. lignano*. Moreover, if one of the adhesive genes is knocked down by RNAi, *M. lignano* is impeded from attaching to the substrate [13]. Likewise, we identified that the expression of *ap1-* and *ap2-like* genes is limited to the adhesive organs in three other *Macrostomum* species, independent from the salinity of the habitat. Additionally, the RNAi knockdown of either or both adhesive genes rendered them unable to attach. These findings indicate that this basic set of two adhesive proteins is enough to function as a reversible glue in all aquatic conditions.

### 3.3. Conserved Domains in Adhesive Proteins

Bioadhesive proteins often contain regions with a conserved protein domain structure. In a recent review, it was pointed out that the combination of the protein domains “von Willebrand factor type D domain (vWD)”, “domain of eight conserved cysteines (C8)”, and “trypsin inhibitor like domain (TIL)” is common in aquatic adhesive proteins [25]. These protein domains were found, for example, in limpets [12], sea stars [37,38], sea urchins [39], the proseriate flatworm *Minona ileanae* [40], and in *M. lignano* [13]. The protein domains vWD, a C8, and a TIL are part of the giant multi-domain “von Willebrand factor” (vWF) protein [41]—an intensely studied protein involved in protein–protein interactions and protein multimerisation [42,43]. The vWF is an evolutionary conserved, very abundant domain that can also be found in mucins, in zonadhesin, in otogelin, and in vitellogenin [44]. Recently, Javitt et al. [45] showed in great detail that vWF of human mucins form polymers that can further assemble to compact filaments. Notably, in all examined *Macrostomum* species, a vWF domain was present in ap1 and ap2 proteins. The sea star *Asterias rubens* produces the adhesive protein Sfp1, which becomes autocatalytically cleaved in four parts, termed as Sfp1 Alpha to Sfp1 Delta [46]. Three of these four subunits contain the above-mentioned vWD-C8-TIL motif. It was shown that the recombinantly produced rSfp1 Delta did not attach to the substrate in seawater conditions but only adsorbed to a glass slide in the presence of 150 mM CaCl_2_ [47]. Although it was shown that the vWD-C8-TIL domain could technically adsorb to a non-protein substrate, it is unlikely that these conditions are met in nature. Thus, we reason that this domain is not the responsible part for surface adsorption in aquatic environments.

The central region of the studied ap1 proteins also holds a C-type lectin-like domain (C-Lect). These domains bind to a variety of ligands. As the name suggests, sugars are the primary target. However, C-Lect domains were shown to also bind to proteins, lipids, or other compounds [48]. Furthermore, another part of the *Macrostomum* ap1 proteins were the variable numbers of EGF domains. It was proposed that the EGF domain in the sea star adhesive protein Sfp1 Beta might play an important role in adhesion in seawater environments [49]. In *M. lignano*, it was suggested that the EGF domains of ap1 assemble into a fibrillin-like protein [13]. The protein-domain organisation of the ap1 proteins in the studied *Macrostomum* species shows high similarity, with the exception of the number of the EGF domains. This difference might be of technical nature due to the short-read-based transcriptome assembly. Likewise, the highly positively charged Lysine–Arginine-rich regions of Mlig-ap1 (indicated as GRK-rich region in Figure 3a), which comprise about two-thirds of the whole protein in *M. lignano*, were only partially identified in the transcriptome assemblies of the *Macrostomum* species of the present study. We suggest that the ap1-like genes in the analysed species most probably also contain an excessive amount of these Lysine–Arginine-rich regions, and they most certainly play a crucial role in temporary adhesion in flatworms. However, these low complexity regions are composed of a low diversity region of amino acid residues, and, therefore, an assembly using short-read data hampers the reconstruction of the respective sequence. The function of this region remains enigmatic in *M. lignano* and the species studied here.

Mlig-ap2, the proposed surface-binding protein of *Macrostomum lignano*, consists of 14.794 amino acids [13]. In the N-terminal end of Mlig-ap2 as well as the ap2a in the analysed species, three different protein domains were present, including TIL domains, C8 domains, and a vWD domain. As mentioned above, we reason that N-terminal ends of the identified Mlig-ap2-like proteins are also not responsible for substrate binding.

At the C-terminal end of Mlig-ap2 and the ap2b fragments in the analysed species, multiple TIL domains and TSP-1 domains were present. TIL domains are cysteine-rich disulfide bridge forming regions that show inhibitory activity against diverse proteases [50]. The protease inhibitory function of TIL domains was reported for many species (reviewed in [51]). There are no reports of an adhesive function of TIL domains. In addition, TSP-1 domains were shown to interact with a broad spectrum of ligands, including extracellular matrix proteins, receptors, growth factors, cytokines, and proteoglycans [52,53]. However, no indications for adherence to inorganic substrates were described for TSP-1 domains. Therefore, an essential contribution to the attachment to substrates of the Mlig-ap2-like TIL and TSP-1 domains of the *Macrostomum* species studied here is unlikely.

Two repetitive motifs constitute nearly three-quarters of the entire ap2 protein in *M. lignano* [13]. Since such a large proportion of this protein consists of those regions, it is likely to be highly significant for the adhesion mechanism. In the other analysed species, we were only able to find fragments of the putative repeat regions 1 and 2 in ap2-like proteins. A difference in these repeat units is certainly important for adhesion and requires more attention. However, solid genomes with good protein predictions are inevitable to investigate these regions further.

### 3.4. Large Adhesive Genes Cannot Be Sequenced with Short-Read Technology

Large stretches of the adhesive proteins Mlig-ap2 are tandem repeats. A single unit of several hundred amino acids is repeated dozens of times [13]. Likewise, in the flatworm *M. ileanae*, the protein Mile-ap2a is repeated at least 12 times in the putative Mile-ap2 protein. In the present work, we were unable to assemble the complete ap2 of all five species. We consistently identified only the N-terminal and C-terminal ends of the respective ap2 proteins, with the central part, probably containing the repeat motifs, missing (Figure 3b). Mlig-ap2 could only be inferred from the complete assembled genome, and the ap2 in *M. ileanae* could only be partially resolved using ultra-long reads produced by Oxford Nanopore sequencing. Similar to this, the full-length spider-silk proteins AgSp1, containing 43 and 38 iterations of a repeat motif 1 and 2, respectively, could only be inferred by using Oxford Nanopore long-read sequencing [7]. Long-read sequencing, e.g., Oxford Nanopore or PacBio sequencing, seems to be an effective approach to obtain the full sequences of the giant adhesive proteins.

### 3.5. Post-Translational Modifications and Their Role in Bioadhesion

Many animals contain similar conserved domain regions in the adhesive proteins. Another important aspect of adhesive proteins might be post-translational modifications (PTMs). For example, the strong adhesive produced by mussels largely relies on a post-translational modified tyrosine to DOPA [54]. Thus far, DOPA has only been detected in permanent adhesives and has not been identified in the secretions of temporary adhering animals. Phosphorylation of serine was observed in the adhesives of sea cucumbers and polychaetes [55,56]. In mussels, limpets, sea stars, and sea cucumbers, sulphated proteins were identified in the secretory cells and adhesive secretions [57]. Glycosylation is another PTM that is often reported in marine bioadhesives. Studies on the sea stars *Asterias rubens* [58] and *Asterina gibbosa* [59], the sea urchin *Paracentrotus lividus* [60], the barnacle *Balanus amphitrite* (now *Amphibalanus amphitrite*) [61], the green mussel *Perna viridis* [62], some ascidian species [63], and the limpets *Lottia limatula* [64] and *Patella vulgata* [12] all report glycosylation in the adhesive cells and/or adhesive proteins. In addition, glycosylation was shown to be the key player in the surface interaction of spider silks [65]. Glycosylated proteins are present in adhesive organs of flatworms, e.g., in the proseriate *Minona ileanae* [40] and in the planarian *Schmidtea mediterranea* [66]. In *M. lignano*, the sugar Gal-β(1–3)-GalNAc was detected in the outer rim of the adhesive vesicles [24] and is part of the glycosylated Mlig-ap2 [13]. In this study, PNA staining in *M. hystrix* and *M. pusillum* showed that the same glycosylation is present in the outer rim of the adhesive vesicles in these two species (see Supplementary Figure S4). This suggests that Mhtx-ap2 and Mpus-ap2 share the same glycosylation as Mlig-ap2. PNA did not stain the adhesive system of the freshwater species *M. tuba*. Notably, we did not observe any lectin staining in *M. tuba* adhesive cells. This could imply that glycosylation of ap2 could be altered in freshwater systems. However, we suggest it might be due to technical limitations.

### 3.6. The Two-Component Flatworm Adhesive could Lead to the Development of Novel Biomimetic Glues

Understanding bioadhesion of aquatic organisms can lead to the development of novel biomimetic adhesives [67,68,69]. Flatworm-based biomimetic adhesives could have much-desired characteristics, including (1) a high adhesive capacity in wet environments, (2) a very rapid attachment, (3) a reduced or complete lack of toxicity or carcinogenesis, (4) no need for petroleum-based raw materials, (5) fast reversibility. In the present study, we compared bioadhesion of flatworms from marine, brackish, and freshwater environments. A basic set of two adhesive proteins was found to be highly similar in all species. Therefore, we propose that Mlig-ap1 and Mlig-ap2 might function in all aquatic environments, regardless of salinity. These two proteins could serve as a blueprint for the development of novel biomimetic adhesives with beneficial properties for medical or industrial wet adhesion requirements.

## 4. Materials and Methods

### 4.1. Description of Sampling Sites of the Fresh, Brackish, and Seawater Species Cultures/Sampling

The freshwater species *Macrostomum poznaniense* was collected from debris sampled from the overflow gutter of a zebrafish facility located at the Institute for Molecular Biology in Innsbruck, Austria (47.26461, 11.34260). Animals were picked individually from the debris under a stereomicroscope. Species determination was performed by live squeeze preparation and observation under a microscope (see also [25]). *M. poznaniense* could only be held for several days in the laboratory at room temperature in filtered water collected from the sampling location.

The freshwater species *Macrostomum tuba* was sampled from a pond in the palmtree house located at the “Hofgarten” in Innsbruck, Austria (47.27291, 11.39662). Sample determination was performed in a similar manner as for *M. poznaniense*. *M. tuba* was cultured in plastic boxes (20 × 30 × 5 cm) at room temperature using Planaria medium [70] with the addition of rotifers or *Paramecium* as a food source two times a week. Notably, *M. tuba* tends to be cannibalistic; therefore, only animals of the same size were kept in one box.

The brackish water species *Macrostomum hystrix* and both seawater species *Macrostomum pusillum* and *Macrostomum spirale* were kept as permanent cultures [71]. *Macrostomum hystrix* was kept at 7‰ artificial seawater (ASW), whereas *M. pusillum* and *M. spirale* were kept at 32‰ ASW. All three species were kept in Petri dishes and fed with the algae *Nitzschia curvilineata* Hustedt 1922 [72] every two weeks.

### 4.2. Documentation of Live Squeeze—Preparations

Animals were anaesthetized (see paragraph below), mounted on a microscope slide in a small droplet of their respective culture medium, and covered with a coverslip equipped with small paraffin spacers on all four corners. Images of live animals were then taken on a Leica DM5000 B microscope equipped with either a Leica DFC 490 or a Leica DFC 495 camera (Leica Microsystems, Wetzlar, Germany).

### 4.3. Anaesthetization and Fixation

The seawater species *M. pusillum* and *M. spirale* were gradually anaesthetized for 40 and 50 min in 7.14% (*w*/*v*) magnesium chloride hexahydrate (MgCl_2_ × 6 H_2_O), respectively. The brackish water species *M. hystrix* was gradually anaesthetized with precooled 0.1% *v*/*v* 1-phenoxy-2-propanol (1P2P) in 7‰ ASW for 45 min. The freshwater species *M. tuba* and *M. poznaniense* were gradually anaesthetized with 10% *v*/*v* ethanol in tap water for seven minutes on ice.

After being anaesthetized, all animals were fixed for one hour in 4% (*w*/*v*) formaldehyde in phosphate-buffered saline (1× PBS) at room temperature. Then, the fixative was removed, and specimens were rinsed at least four times over the course of 20 min with 1× PBS with 0.1% (*v*/*v*) Tween (PBSw). An ascending methanol series was performed (25%, 50%, 75%, 100%, 100%, 100%; methanol in 1× PBS) prior to being stored at −20 °C.

### 4.4. RNA Extraction, cDNA Synthesis, Template Synthesis by PCR, and Probe Synthesis

Total RNA was extracted from all animals using TRI reagent^®^ (Sigma-Aldrich, St. Louis, MO, USA) (see following Section 4.5 for exact protocol) and cDNA was synthesised according to the manufacturer’s instructions (SuperScript IV First-Strand cDNA Synthesis, Thermo Fisher Scientific, Waltham, MA, USA) with either oligo-dT or random hexamers.

For all five *Macrostomum* species, primers were designed with primer3 [73] for the *Mlig-ap1-*, *Mlig-ap2-*, and *Mlig-if1-like* transcripts (see Supplementary Table S5). These homologues were identified by a BLAST search against their respective transcriptomes (see Supplementary Table S5). SP6 polymerase (5′-CATTTAGGTGACACTATAGAAG) and T7 polymerase (5′-GGATCCTAATACGACTCACTATAGG[G]) promoters were added to the forward and reverse primers, respectively. Polymerase chain reaction (PCR) was carried out in 50 µL, and the conditions were 30 s at 98 °C for initial denaturation, followed by 35 cycles (10 s at 98 °C, 30 s at 56 °C, 30 s at 72 °C), a final elongation for 2 min at 72 °C, and stored at 4 °C. PCR products were cleaned up using the Roche HighPure PCR cleanup kit (Roche, Basel, Switzerland) and eluted in 30 µL elution buffer. If multiple bands were visible in a 1% agarose gel, the bands were extracted using the Monarch DNA Gel Extraction Kit (New England Biolabs, Ipswich, MA, USA) and were eluted in 10 µL elution buffer. A re-PCR of the extracted bands was performed with similar PCR conditions as mentioned above, except with only 25 cycles.

### 4.5. RNA Extraction and Sequencing of Macrostomum Tuba

Ten adult egg-bearing *M. tuba* were collected and were individually checked by squeeze preparation for species determination. Total RNA was extracted with TRI reagent^®^ (Sigma-Aldrich, St. Louis, MO, USA) from the ten pooled animals. The animals were slowly pipetted up and down 20 times in 1 mL TRI reagent^®^. Then, 200 µL chloroform was added, and the solution was pulse-vortexed for 15 s and incubated at room temperature for 15 min. The tube was then centrifuged for 20 min at 12,000× *g* at 4 °C. The upper phase was recovered and was mixed with 500 µL isopropanol in a new tube and incubated for 10 min at room temperature. After another centrifugation step for 10 min at 12,000× *g* at 4 °C, the supernatant was removed. The pellet was washed two times with 1 mL 75% ethanol until the pellet floated and centrifuged two times for 5 min at 7500× *g* at 4 °C. Finally, all supernatant was removed, the pellet was air-dried for 10 min prior to resuspending it in 20 µL nuclease-free water. The resuspended RNA was then stored at −80 °C. RNA was sent for library preparation (NEBNext^®^ Ultra™ II Directional RNA Library Prep Kit for Illumina, New England Biolabs, Ipswich, MA, USA) to Vienna BioCenter (Vienna, Austria). The following adapters were used for Illumina sequencing: NEB: 7004:UD7004:TGTTGATC 5004:UD5004:CTATGTTA. Sequencing (paired end 150 bp) was carried out on a NextSeq550 system (Illumina, San Diego, CA, USA) and resulted in a total of 38,081,193 PE150 reads.

### 4.6. Transcriptome Assembly and Annotation of Macrostomum Tuba

Prior to the assembly, we applied a pipeline onto the raw reads to correct and trim away low-quality bases. Here, we used fastqc v0.11.9 (https://www.bioinformatics.babraham.ac.uk/projects/fastqc/, accessed on 18 October 2021) to assess the quality of the raw reads; corrected the raw reads with rcorrector [74] (commit ce5d06b), dependent on jellyfish v.2.2.8 [75] using default settings; checked if both mates are present with the script “FilterUncorrectabledPEfastq.py” from TranscriptomeAssemblyTools (https://github.com/harvardinformatics/TranscriptomeAssemblyTools, accessed on 18 October 2021) (commit e2df226); and trimmed Illumina adapters with TrimGalore v0.6.4_dev (https://github.com/FelixKrueger/TrimGalore, accessed on 18 October 2021), dependent on Cutadapt v1.15 [76], with the following flags: “--paired --retain_unpaired --phred33 --length 36 -q 5 --stringency 1 -e 0.1 --cores 14 --gzip”. Pigz v2.4 was used to allow for multicore processing of gzip compression steps. The corrected and trimmed reads were then assembled using Trinity v2.11.0 [77] with the flags “--SS_lib_type RF --no_salmon --max_memory 60G --CPU 31”.

The completeness of the assembled transcriptome was assessed with BUSCO v.4.0.6 [78], dependent on Augustus v.3.3.3 [79], Prodigal v.2.6.3, sepp commit bd26318, hmmer v.3.3, biopython v.1.76, blast v.2.10.0+, and R v.3.6.2, using mezazoa_odb10 database. We annotated the transcriptome using Trinotate v.3.2.0, which is dependent on TransDecoder v.5.5.0, SQLite v.3.22.0, hmmer v.3.3, hmmersearch v.2, signalp v.4.1 [80], and tmhmm v.2.0c. The script “TransDecoder.Predict” from the TransDecoder pipeline was run with the flag “--single_best_only”.

### 4.7. Transcriptome Data of M. pusillum, M. spirale, M. hystrix, and M. poznaniense

We accessed the annotated transcriptomes for the marine species *M. pusillum* and *M. spirale*, as well as the brackish water species *M. hystrix* from a previous publication [71]. In addition, we recently published the transcriptome of the freshwater species *M. poznaniense* [25]. All four transcriptomes are publicly available (https://doi.org/10.5281/zenodo.3547572, accessed on 18 October 2021; https://www.ncbi.nlm.nih.gov/nuccore/GIJT00000000, accessed on 18 October 2021).

### 4.8. BLAST Searches and Conserved Domain Identification

All five transcriptomes were added to separate BLAST v.2.10.0+ databases and were deployed on a sequenceserver v.2.0.0.4rc instance running at a local Linux workstation [81]. Transcripts with the highest score were translated into an amino acid sequence and submitted to NCBI conserved domain search with default settings on the conserved domain database (CDD) v.3.19 [82].

### 4.9. In Situ Hybridisation

In situ hybridisation for the freshwater species *M. tuba* was performed using the previously published protocol for *M. lignano* [13,83]. The other species (*M. hystrix* and *M. pusillum*) were treated with a slightly modified version of the *ISH* protocol from King and Newmark [84]. We fixed the animals as mentioned in the previous Section 4.3, and we omitted the formamide bleaching step. Probe concentration was 0.15 ng/uL at 56 °C overnight, shaking at 350 rpm. Blocking was performed for 3 h at 4 °C, and the antibody was diluted at 1:4000 and incubated overnight at 4 °C. All steps were carried out in 1 mL volume, except the antibody incubation step (700 µL) as well as the colour development step (500 µL).

### 4.10. Double-Stranded RNA Synthesis and RNAi Adhesion Assay

Synthesis of double-stranded RNA specific for the gene knockdown was carried out in a total volume of 25 µL or 20 µL using the HighScribe SP6 and HighScribe T7 RNA synthesis kits (New England Biolabs, Ipswich, MA, USA), respectively, according to the manufacturer’s protocol. In short, the ssRNA produced by SP6 and the ssRNA produced by T7 were mixed, heated to 70 °C for 5 min, and then slowly cooled down to room temperature, wrapped in multiple layers of aluminium foil for double-stranded RNA formation. An amount of 2 µL of 1:200 diluted RNAse A, as well as 2 µL of DNAse (Roche, Basel, Switzerland), was added to degrade single RNA strands as well as template DNA, and the reaction was incubated for 30 min at 37 °C. Alcohol precipitation was performed with 4.9 µL 3 M sodium acetate and 49 µL isopropanol. The reaction was incubated for 5 min at RT before being centrifuged at maximum speed for 30 min at 4 °C. The supernatant was removed, the pellet washed with 500 µL precooled 75% ethanol, and centrifuged again for 5 min at 7400× *g* at 4 °C. The supernatant was again removed, and the pellet was air dried for up to 10 min. We diluted the dsRNA with 100 µL nuclease-free water, and checked the quality and quantity of an agarose gel. dsRNA was aliquoted in 4 µL batches and stored at −80 °C. RNAi was performed by soaking the animals in a mixture of ASW, algae, dsRNA, and antibiotics. This soaking solution was changed every day with a rotation of three different antibiotics (kanamycin, streptomycin, and ampicillin). An off-target dsRNA control was performed with dsRNA against the firefly luciferase (pGEM-*luc* Vector, Promega, Madison, WI, USA) in *M. tuba*.

The adhesion assay was carried out by up to five independent investigator-blinded researchers that quantified the number of attachments during 60 s. Each investigator analysed four (*M. pusillum*) or two (*M. hystrix*) individuals for three different conditions: control animal without dsDNA treatment, dsRNA against *ap1*, and dsRNA against *ap2*. For *M. tuba*, a total of 12, 11, and 12 animals were observed by three investigators for dsRNA against *ap1*, dsRNA against *ap2*, and control animals, respectively. The figure with statistical analyses was performed in R v. 3.6.3 using the libraries rstatix v0.7.0, ggpubr v0.4.0, and ggplot2 v3.3.4. The digits above the graphs is the *p*-value of a *t*.test, invoked by “method = *t*.test” in the method “stat_compare_means”.

### 4.11. Attachment Assay in Different Salt Concentrations

We assessed if animals were still able to attach to the embryo dish or to the inside of the pipet tip in different salt concentrations. We transferred five adult specimens of *M. tuba*, *M. hystrix*, *M. spirale*, and *M. pusillum* in a glass embryo dish and gradually changed the salt concentrations over the course of 2.5 h. Adhesion capabilities were tested at different salt concentrations: in deionized water containing 2 parts per million salt (ppm), 5 ppm, 10 ppm, 20 ppm, 35 ppm, 45 ppm, and 60 ppm. *M. tuba* started in fresh water and was gradually submitted up to saltwater concentrations of 5 ppm. *M. hystrix* started at 7 ppm, and salt concentrations were gradually lowered to 2 ppm, and then risen to 45 ppm. *M. pusillum* and *M. spirale* started at 32 ppm, and salt concentration was gradually risen up to 60 ppm and then gradually lowered to 2 ppm.

### 4.12. Lectin Staining and gSTED High-Resolution Microscopy

Lectin stainings and imaging were performed according to Lengerer et al. [24]. In short, specimens were stained with biotinylated peanut agglutinin and streptavidin Dylight 488 and mounted in Mowiol. Samples were analysed with an SP8 gSTED high-resolution microscope (Leica Microsystems, Wetzlar, Germany). Acquired images were deconvolved using a Huygens professional software (Scientific Volume Imaging, Hilversum, The Netherlands).

### 4.13. Transmission Electron Microscopy

Animals were anaesthetized as mentioned above and chemically fixed by using two different, specifically tailored protocols, depending on the animals’ habitat [85]. The freshwater species *M. poznaniense* and *M. tuba* and the brackish water species *M. hystrix* were fixed on ice according to Rombout et al. [86], whereas the marine species *M. spirale* and *M. pusillum* were best preserved by fixation according to Eisenmann and Alfert [87]. After washing with a cacodylate buffer, samples were dehydrated in an increasing series of acetone and embedded in EMBed 812 epoxy resin (Science Services, Munich, Germany). As a complementary approach, *M. spirale* and *M. lignano* were high-pressure frozen, freeze-substituted and embedded in EMBed 812 [85]. Ultrathin sections were stained with lead citrate and examined with a Zeiss Libra 120 energy filter transmission electron microscope (EFTEM) (Zeiss, Oberkochen, Germany). Images were made with the ImageSP software and a high-speed 2 × 2 k camera (Tröndle, Moorenweis, Germany). Note that the general ultrastructure of the adhesive organs was equally well preserved in all species investigated, irrespective of the fixation method used.

### 4.14. Element Analysis with EELS and ESI

Electron energy loss spectroscopy (EELS) and electron spectroscopic imaging (ESI) were used for element analysis with a Zeiss Libra 120 EFTEM (Zeiss, Oberkochen, Germany). Nitrogen (N), as a leading element of proteins and glycoproteins, was measured spectroscopically with parallel EELS by using its energy loss at 397 eV at the N-K edge. ESI was performed using a three-window method and the ImageSP software. A high contrast image made at 250 eV was inverted, combined with the maximum element distribution, and mix-mapped with false colours.

### 4.15. Serial Block-Face Scanning Electron Microscopy

Serial block-face scanning electron microscopy (SBFSEM) is a method where serial sectioning of a specimen is performed by an ultramicrotome, which is located in the specimen chamber of a scanning electron microscope (SEM) [88]. After each cut, an SEM image of the specimen’s block-face is stored, leading to a series of images, which enable a three-dimensional (3D) reconstruction of a volume of interest. In this work, an ESEM Quanta 600 FEG equipped with a Schottky emitter (FEI, Eindhoven, The Netherlands) was used. SBFSEM was performed with a 3View system from Gatan (Gatan, Pleasanton, CA, USA) [89,90]. Imaging was realised with a backscattered electron (BSE) detector from Gatan at an electron energy of 3 keV. In order to avoid charging on the specimen´s surface, the work was carried out in the low vacuum mode of the ESEM using water vapor as an imaging gas. According to a slice thickness of 100 nm, a voxel size of 5 × 5 × 100 nm^3^ was achieved. Samples for SBFSEM were fixed according to the protocol of Deerinck et al. [91] using glutaraldehyde, ferrocyanide osmium tetroxide postfixation, thiohydrocarbazide-osmium liganding and uranyl acetate, and lead aspartate en bloc staining to enhance contrast for BSE imaging.

### 4.16. Reconstruction of the Adhesive System

A total of 441 images (Supplementary Materials available through an online repository, see “Data Availability Statement”) were imported into ORS DragonFly v. 2021.1 and aligned using the “sum of squared differences” algorithm, with the setting “initial step” increased to 42% for both the *x*- and *y*-axis. Segmentation was performed in a semi-automatic way by outlining the cell boundaries with the “2D round brush” on every fifth to the tenth section and filled in automatically. An automatic Z-interpolation step with manual corrections was performed. The microvilli were individually outlined with the “path tool”. The releasing and adhesive vesicles in the reconstruction were manually drawn with the “3D brush” but do not represent the exact location in the image. However, care was taken to keep the ratios between both vesicle types. In order to build the 3D reconstruction, the outlined regions of interest in the stack were transformed into meshes and smoothened independently: anchor cell (7 times/1 iteration), releasing gland (11/1), adhesive gland (15/1), epidermis (10/1), releasing vesicles (0/1), and adhesive vesicles (3/1). The movie of the 3D model was generated with the included “movie-maker” function.

### 4.17. Figure and Movie Preparation

Figures were assembled using the open-source tools GIMP v2.10.24 and Inkscape v. 1.1. The background around the living animals of Figure 1 and Figure 4 was partially filled by using the “smudge” tool included in GIMP. Fiji [92], built on ImageJ v. 1.52p, was used to create confocal projections of the lectin-stained tail plates. Kdenlive v. 17.12.3 was used to create the supplementary movie of the adhesive organ reconstruction.

## Figures and Tables

**Table 1:** Adhesion assay in different salt concentrations.

	Salt Concentrations [ppm]						
Species	2	5	10	20	35	45	60
*M. pusillum*	+++	+++	+++	+++	+++	+++	+++
*M. spirale*	+++	+++	+++	+++	+++	+++	+++
*M. hystrix*	+++	+++	+++	+++	+++	x	x
*M. tuba*	+++	x	x	x	x	x	x

+++ = attachment confirmed. x = animals showed aberrant behavior or deceased, and adhesion could not be tested.

## Data Availability

The transcripts were deployed at NCBI with the following accession numbers: Mpus-ap1 (OK245455), Mpus-ap2a (OK245456), Mpus-ap2b (OK245457), Mpus-if1 (OK245458), Mspi-ap1 (OK245459), Mspi-ap2a (OK245460), Mspi-ap2b (OK245461), Mspi-if1 (OK245462), Mtub-ap1 (OK245463), Mtub-ap2a (OK245464), Mtub-ap2b (OK245465), Mtub-if1 (OK245466), Mhtx-ap1 (OK245467), Mhtx-ap2a (OK245468), Mhtx-ap2b (OK245469), and Mhtx-if1 (OK245470). The PE150 Illumina reads from *Macrostomum tuba* as well as the assembled and annotated transcriptome, all 441 images used for *M. lignano* adhesive organ reconstruction, and the data of the RNA interference assay are openly available in a zenodo repository at https://doi.org/10.5281/zenodo.5519227 (accessed on 18 October 2021), Version 1.

## Supplementary Materials

The following are available online at https://www.mdpi.com/article/10.3390/ijms222212228/s1.
