# Supplementary material for: (Un)expected Similarity of the Temporary Adhesive Systems of Marine, Brackish, and Freshwater Flatworms
Source: Int J Mol Sci. 2021 Nov 12;22(22):12228. doi: 10.3390/ijms222212228 (PMC8621496; doi:10.3390/ijms222212228)
Supplement: Supplementary file 1 [file ijms-22-12228-s001.zip › ijms-1448713-supplementary file legends.pdf]

Supplementary video S1: Movie of the 3D reconstruction using SBFSEM data.

Supplementary Figure S2: Element spectrographic imaging analysis for nitrogen content in the adhesive vesicles. Marine species outlined in pink, brackish species outlined in yellow, freshwater species outlined in green. Scale bar: 200 nm.

Supplementary Figure S3: Alignments of ap1-like and ap2-like proteins to *Macrostomum lignano* ap1 and ap2, respectively. The protein sequences for *M. poznaniense*, *M. tuba*, *M. hystrix*, *M. pusillum*, and *M. spirale* were used as subjects, and the protein sequence of *M. lignano* was used as a BLAST query. The table shows the coverage percentage of the query sequence (Cov. [%]), the E-value (E-val.), and the percentage on how identical the subject is to the query (Ident. [%]). In ap2-like BLAST, a fragment of the repeat motif 1 in ap2a and a fragment of repeat motif 2 in ap2b mapped to the RP-1 and RP-2 regions of *M. lignano*, respectively. Notably, this way, the number of mapped repeats is only corresponding to *M. lignano* and does represent the number of repeats in the other species.

Supplementary Figure S4: Superresolution images of PNA lectin stained tail plates of *M. hystrix* and *M. pusillum*. The cell bodies of the adhesive gland cells of *M. hystrix* (**a**) and *M. pusillum* (**b**) and their respective gland cell necks elongate towards the tip of the tail (**c, d**). Only the outer rim of the adhesive vesicles was stained by PNA, which results in donut-shaped structures. This stained outer rim most probably corresponds to the glycosylated ap2 adhesive protein (light outer rim in transmission electron images). Note: no particular orientation of the animal is given for this figure. Scale bar: 2 µm.

Supplementary Table S5: Table containing the primers and sequencing data of ap1, ap2a, ap2b, and if1 that were used in this work.

Supplementary Figure S6: Whole-mount in situ hybridisation of *ap1-* and *ap2-like* genes in three *Macrostomum* species with the Sense-control probes (negative control). Control whole-mount in situ hybridisation of *ap1-* and *ap2-like* genes in three Macrostomum species. *Ap1* and *ap2* expression in the marine *M. pusillum* (**a,b**), in the brackish water *M. hystrix* (**c,d**), and in the freshwater *M. tuba* (**e,f**). Marine species outlined in pink, brackish species outlined in yellow, freshwater species outlined in green. (**e**) Sense control of a different Mtub-ap1 region than shown with the Mtub-ap1 antisense probe. Scale bars 50 µm in (**a, b**); 100 µm in (**c**–**f**).
