# Supplementary figures and images for: (Un)expected Similarity of the Temporary Adhesive Systems of Marine, Brackish, and Freshwater Flatworms
Source: Int J Mol Sci. 2021 Nov 12;22(22):12228. doi: 10.3390/ijms222212228 (PMC8621496; doi:10.3390/ijms222212228)

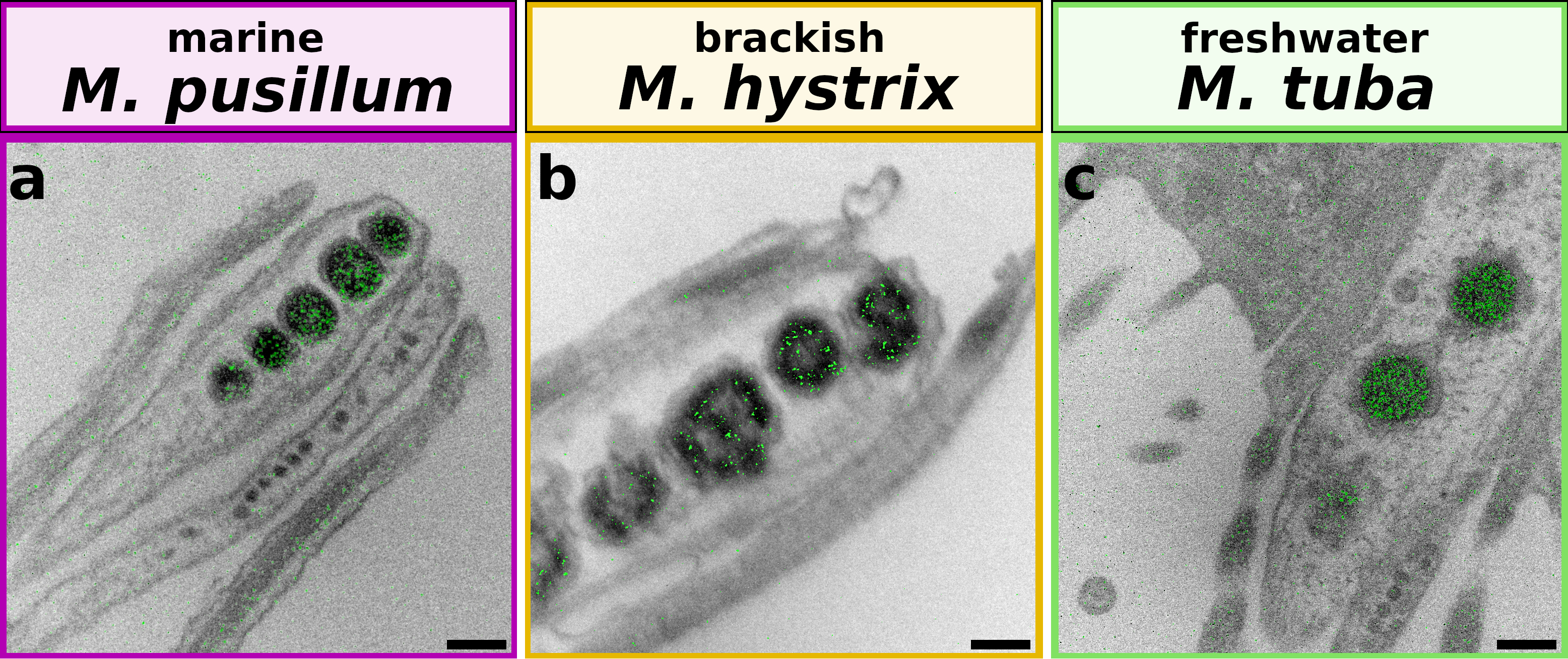

Supplement: Supplementary file 1 [file ijms-22-12228-s001.zip › Supplementary_S2.jpeg]

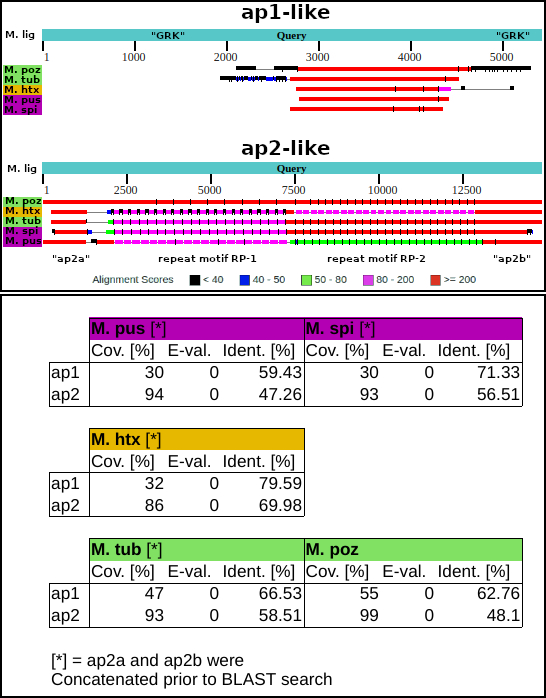

Supplement: Supplementary file 1 [file ijms-22-12228-s001.zip › Supplementary_S3.jpeg]

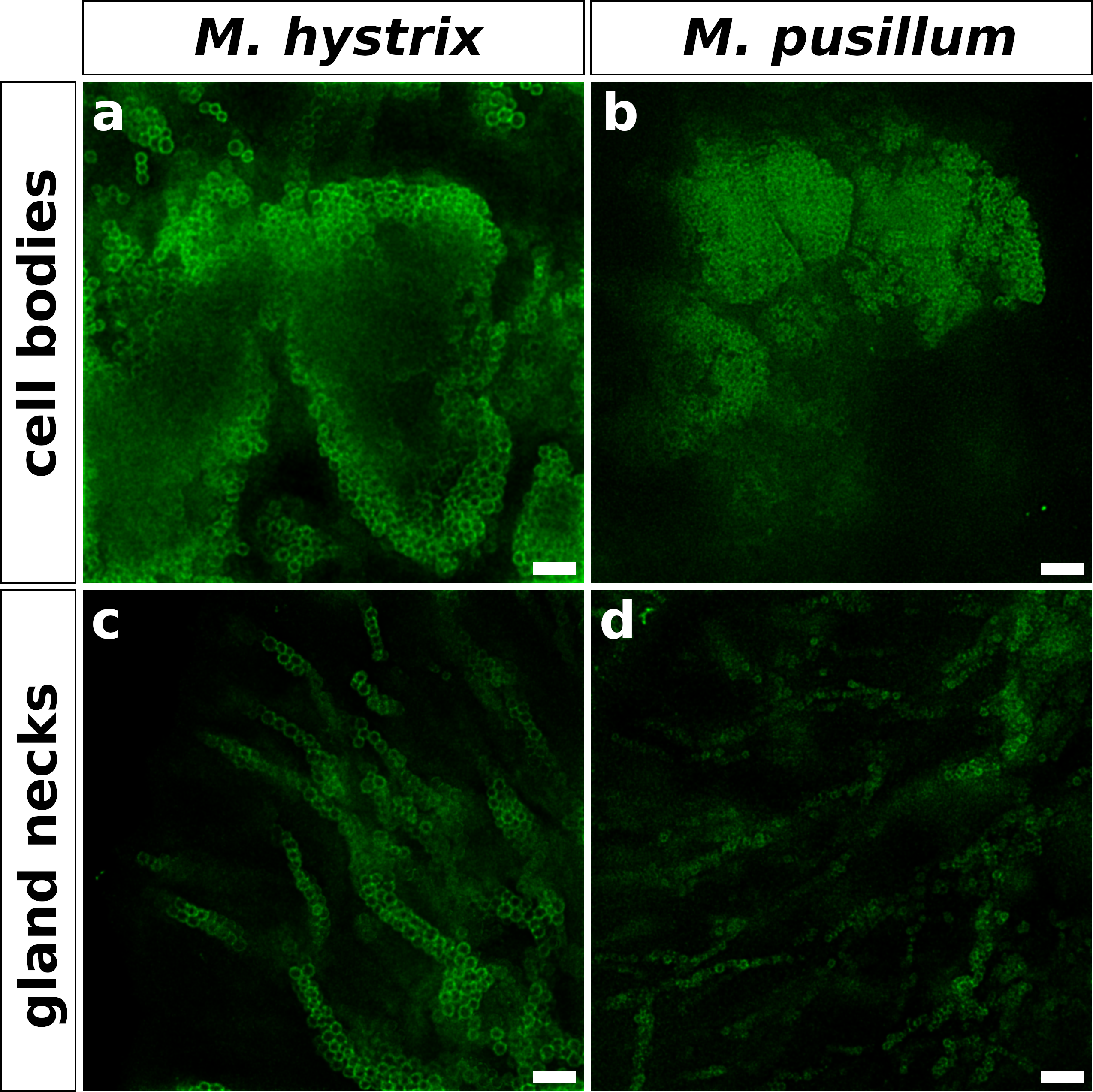

Supplement: Supplementary file 1 [file ijms-22-12228-s001.zip › Supplementary_S4.png]

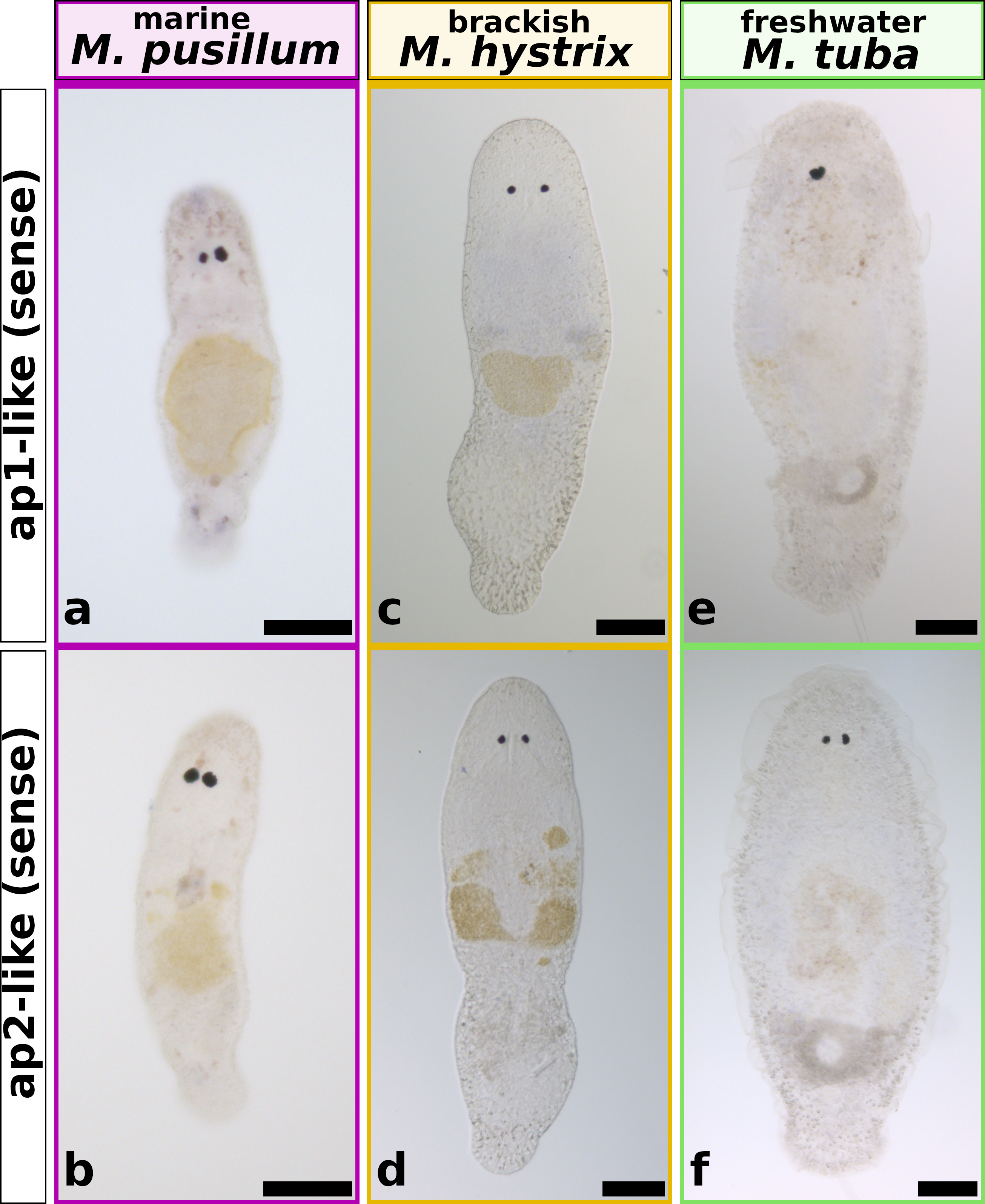

Supplement: Supplementary file 1 [file ijms-22-12228-s001.zip › Supplementary_S6.jpeg]
